# Supradiaphragmatic Jejunal Perforation Following Total Gastrectomy With Esophagojejunostomy Reconstruction for Gastric Adenocarcinoma

**DOI:** 10.7759/cureus.58587

**Published:** 2024-04-19

**Authors:** Ahmed Eldeib, Omar Eldeib, Abdullah Alshammari, Mohammad Aburahmah

**Affiliations:** 1 Department of Surgery, State University of New York (SUNY) Downstate Health Sciences University College of Medicine, Brooklyn, USA; 2 Department of Medical Education, Alfaisal University College of Medicine, Riyadh, SAU; 3 Department of Surgery, Alfaisal University College of Medicine, Riyadh, SAU; 4 Department of Surgery, King Faisal Specialist Hospital and Research Centre, Riyadh, SAU

**Keywords:** nasogastric tube (ngt), left-sided pleural effusion, postoperative complication, jejunal perforation, gastrectomy

## Abstract

Nasogastric tube decompression is a common technique used after abdominal surgery as it is widely accepted to play a role in the management of postoperative ileus and possibly reduce anastomotic leaks after gastrointestinal surgery. However, the routine practice of nasogastric/nasoenteric tube decompression in elective abdominal surgeries has been challenged due to the increased incidence of pulmonary complications and the argued lack of expected benefit. Here, we present a rare complication of nasogastric tube drainage following a routine total gastrectomy for signet-ring cell adenocarcinoma of the cardia in a 43-year-old female. Her postoperative course was complicated with a supradiaphragmatic jejunal perforation presumably from nasogastric tube decompression resulting in a left pleural effusion. The workup included an endoscopy showing the perforation, after which the nasojejunal tube was removed and the patient was managed conservatively. She was eventually discharged on postoperative day 28.

## Introduction

Nasogastric tube decompression is a common technique used after abdominal surgery as it is widely accepted to help play a role in the management of postoperative ileus and possibly reduce anastomotic leaks after gastrointestinal surgery [1]. Nevertheless, nasogastric tube placement can lead to several complications, including strictures, mechanical ulceration, pneumothorax, and perforation [2,3]. This has led many researchers to question the routine practice of nasogastric tube decompression in elective abdominal surgeries due to the increased incidence of pulmonary complications, with several studies concluding that it is no longer reasonable after elective abdominal surgeries [4,5].

Jejunal perforation is an uncommon manifestation of the surgical abdomen. Jejunal perforation has been reported following blunt abdominal trauma, jejunal schistosomiasis, diverticulitis, and abdominal typhoid and milliary tuberculosis [6-8]. Here, we present the case of a jejunal perforation following nasojejunal tube (NJT) placement post-laparoscopic total gastrectomy with D2 lymph node resection. To our knowledge, this occurrence has been rarely reported in the literature with differences in comparison to our report [9-11].

## Case presentation

A 43-year-old female was admitted as a referred case of gastric cancer, signet-ring-cell adenocarcinoma of the cardia, for total gastrectomy. The patient had a history of hypothyroidism, chronic gastritis, and remitted nasopharyngeal carcinoma with no evidence of relapse following neoadjuvant chemotherapy and radiotherapy. Upon admission, the physical examination revealed an alert and vitally stable patient with unremarkable physical findings. Laboratory findings, including complete blood count, basal metabolic profile, and liver function test, were within normal limits.

The patient underwent laparoscopic total gastrectomy with D2 lymph node resection, Roux-en-Y reconstruction with esophagojejunostomy, and jejunojejunostomy with bilateral Jackson-Pratt (JP) drains at the anastomotic sites. NJT was inserted under endoscopic visualization by the anesthesiologist, and its successful placement was verified intraoperatively. No intra or perioperative complications were observed. The patient was kept nil per os (NPO), with intravenous fluids, and NJT attached to low intermittent wall suction.

**Figure 1:**
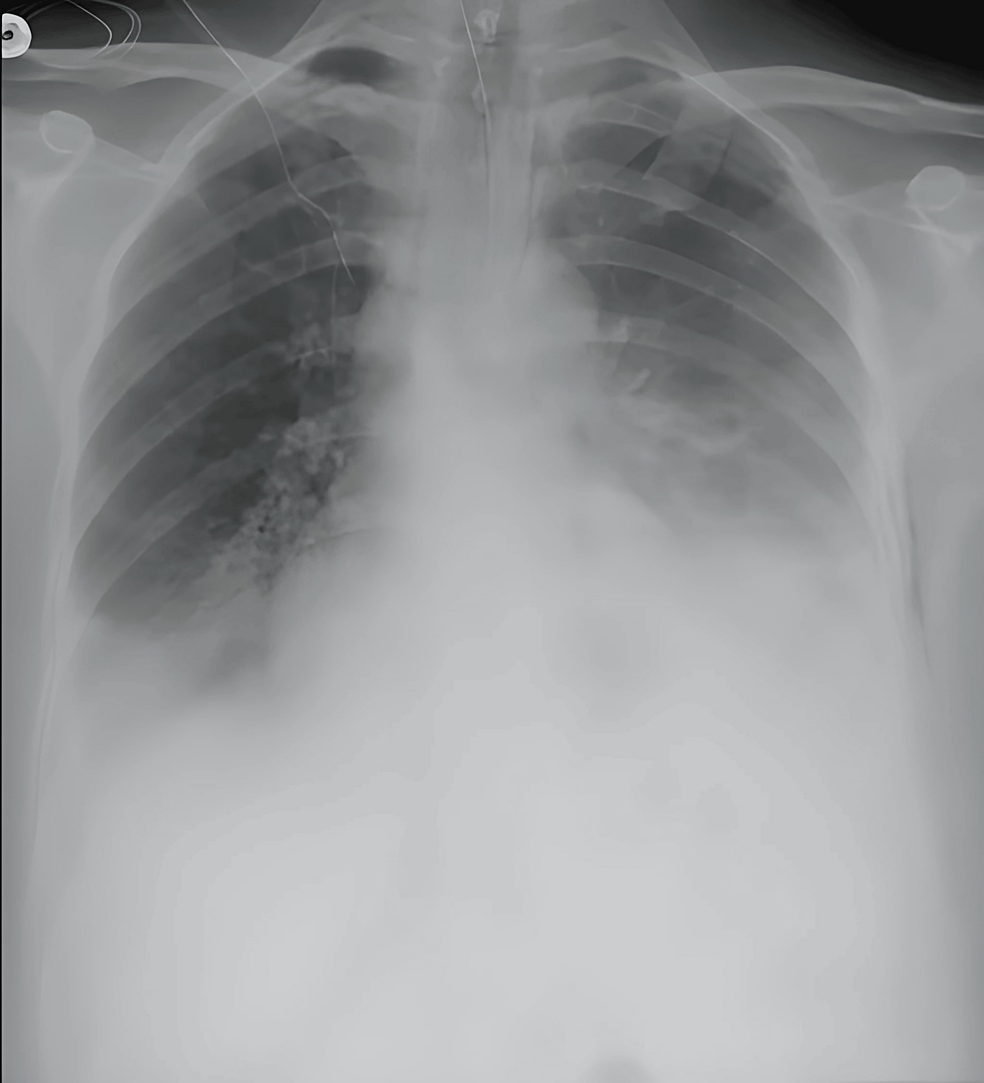
Chest X-ray showing left pleural effusion with pneumomediastinum.

On postoperative day (POD) two, the patient complained of shortness of breath associated with left-sided pleuritic chest pain, along with tachycardia and tachypnea. Chest X-ray showed left pleural effusion with pneumomediastinum (Figure 1).

Pulmonary computerized tomography (CT) angiogram showed a filling defect in the left anterior lower lobe pulmonary artery denoting a pulmonary embolism as well as bilateral pleural effusion associated with pneumothorax more prominent on the left side. Therapeutic anticoagulation using heparin was started. The patient underwent ultrasound-guided pigtail thoracentesis, draining 600 cc of serosanguinous fluid which was sent for workup. The patient was started on empiric broad-spectrum antibiotics.

**Figure 2:**
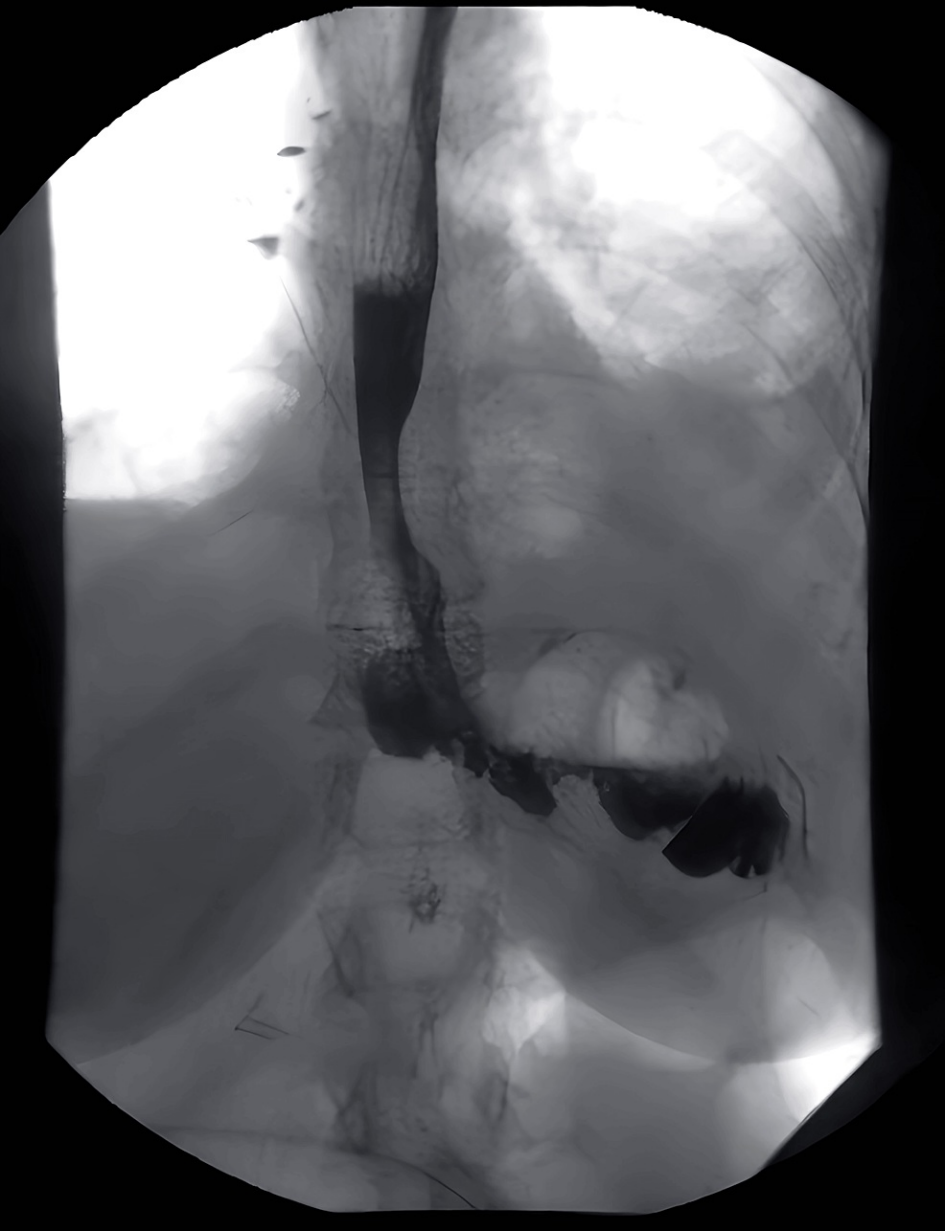
Contrast swallow performed on postoperative day three showing no signs of anastomotic leak with the prompt passage of contrast.

**Figure 3:**
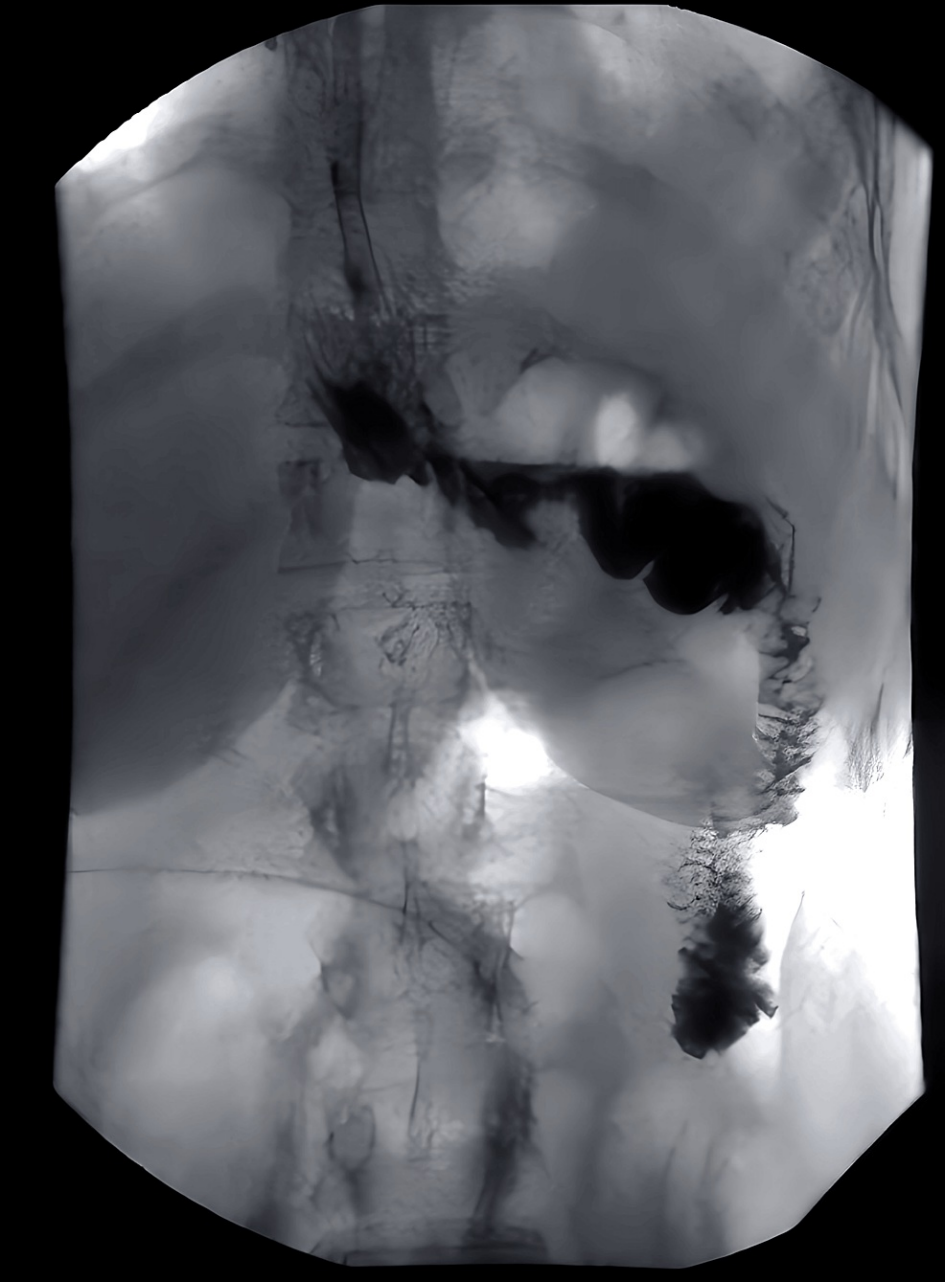
Contrast swallow performed on postoperative day three showing no signs of anastomotic leak with the prompt passage of contrast. The delayed image shows contrast in the small bowel.

Contrast swallow performed on POD three showed no signs of anastomotic leak with the prompt passage of contrast in the small bowel (Figures 2, 3). The NJT tip was visualized in a satisfactory position beyond the anastomosis site.

On POD four, a left-sided chest tube was placed for persistent left-sided pleural effusion which drained copious amounts of brownish fluid concerning for bile and small bowel contents. Fluid culture was positive for gram-negative rods, treated according to susceptibility.

On POD five, chest tube drainage was almost 1,000 cc, and fluid chemistry was positive for both amylase and bilirubin, confirming the intraluminal origin of the fluid. The primary concern at the time was an esophagojejunal anastomotic leak.

**Figure 4:**
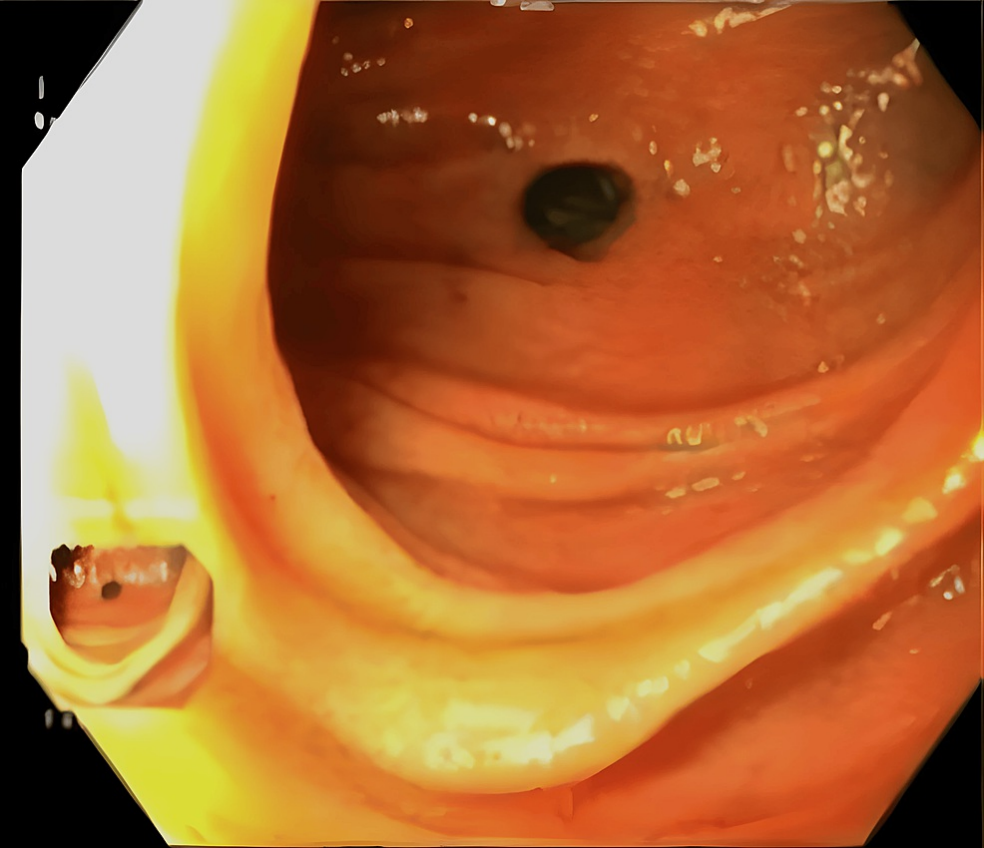
Supradiaphragmatic jejunal perforation on endoscopy.

The patient then underwent a diagnostic endoscopy, which revealed an intact esophagojejunal anastomosis and a jejunal perforation measuring 2 cm in diameter in the segment between the esophagojejunostomy and the jejunojejunostomy (Figure 4).

The perforated viscus was draining above the diaphragm. A methylene blue study confirmed the integrity of the esophagojejunal anastomosis. The NJT tube was subsequently removed. At this point, the observed leak was thought to be secondary to an iatrogenic jejunal perforation most likely caused by the NJT. The patient continued to be vitally stable, and it was decided to manage the perforation conservatively with watchful waiting, NPO, and total parenteral nutrition.

Repeat chest CT with oral contrast performed on POD 20 ruled out residual jejunal leak, after which the patient started oral intake. Her diet was advanced as tolerated, and she was eventually discharged on POD 28.

## Discussion

In an otherwise healthy patient, total gastrectomy and D2 lymph node resection with esophagojejunostomy reconstruction are indicated in stage IB-III gastric cancer [12]. NJT can prove helpful both operatively and postoperatively after total gastrectomy. For instance, a common intraoperative technique is the utilization of an NJT to guide the creation of esophagojejunal anastomosis [13].

Furthermore, NJT placement is utilized for draining postoperative secretions and protecting anastomotic sites, especially after D2 complete lymphadenectomy. The reasoning for this is that the sectioning of sympathetic and parasympathetic nerve fibers during skeletonization of the celiac axis and truncal vagotomy may severely impair intestinal motility [1,14]. Although there is no consensus on the efficacy of its use, NJT placement following total gastrectomy is a fairly common practice among many surgeons [15].

Previous studies have shown that nasogastric/nasojejunal decompression after gastrointestinal surgery may result in an increased incidence of pulmonary complications, including atelectasis and pneumonia [4,15-17]. This holds true in our case as our patient developed bilateral atelectasis and a considerable left-sided pleural effusion. However, this might be unrelated as she also developed a pulmonary embolism.

Anastomotic leakage is one of the known complications of total gastrectomy [9]. The source of leakage is more commonly the esophagojejunal anastomosis. Suspicion of anastomotic complications is usually confirmed using oral contrast studies. An oral contrast study performed on our patient in the early postoperative course revealed no evidence of leakage at the esophagojejunal or jejunojejunal anastomoses. Furthermore, there was no evidence of stenosis. Moreover, daily inspections of intra-abdominal JP drain fluid revealed only serosanguinous fluid, and no suspicion was raised for an early anastomotic leakage. A review of the literature including a Cochrane database review involving more than 5,000 patients showed that the routine use of nasogastric tube decompression after major gastrointestinal surgery might not have any protective value against anastomotic leaks [18]. This has also been suggested by Doglietto et al. in their randomized prospective study on more than 200 patients undergoing total gastrectomy with Roux-en-Y esophagojejunostomy where half of the patients had nasogastric tubes placed and the rest did not. The study showed that the rates of anastomotic leak were similar in both groups [14]. Furthermore, the study showed no difference in the rates of major postoperative and overall postoperative mortality [14,15].

The left-sided pleural effusion that complicated our patient’s postoperative course was not caused by an anastomotic leakage but instead caused by jejunal perforation above the diaphragm. This was confirmed by the contrast swallow study that showed intact esophagojejunal and jejunojejunal anastomoses and the endoscopic visualization of the 2 cm perforation at the site between both anastomoses. Although perforation is a known complication of nasogastric tube placement, to our knowledge, jejunal perforation from NJT placement post-gastrectomy has been rarely reported in the literature. Budisin et al., in their retrospective study of 76 post-gastrectomy for gastric cancer patients, reported an NJT-associated jejunal perforation after the patient developed peritonitis and metabolic acidosis on POD four. The patient was re-operated for suspected anastomotic leakage but was found to have jejunal perforation intraoperatively [9]. Compared to our patient the main differences were that the perforation was found 15 cm distal to the anastomosis where the tube was protruding into the abdominal cavity and that their perforation was subdiaphragmatic instead of supradiaphragmatic in our patient. Other studies have reported infradiaphragmatic jejunal/intestinal perforations as well [10,11].

This case represents an unusual occurrence worth reporting. This also challenges the dogma of nasogastric/nasojejunal tube decompression used for anastomotic protection and is in line with the previous recommendation that the routine placement of nasogastric/nasojejunal tube after Roux-en-Y esophagojejunostomy might be unnecessary in elective total gastrectomy for gastric cancer [14,19].

## Conclusions

We present an unusual case of a supradiaphragmatic jejunal perforation in a 43-year-old female post-total gastrectomy and D2 lymph node resection with Roux-en-Y esophagojejunostomy for gastric cancer. We theorize that the supradiaphragmatic perforation and the subsequent complications were the result of the NJT. Knowledge of this complication, its cause, and this unusual site of perforation leading to an atypical presentation is important not only for prompt diagnosis but also for potential avoidance.
